# Is the role of Health Extension Workers in the delivery of maternal and child health care services a significant attribute? The case of Dale district, southern Ethiopia

**DOI:** 10.1186/s12913-017-2590-8

**Published:** 2017-09-11

**Authors:** Abel Negussie, Gedion Girma

**Affiliations:** Yirgalem Hospital Medical College, Yirgalem, Ethiopia

**Keywords:** Health Extension Workers, MCH service utilization, Southern Ethiopia

## Abstract

**Background:**

The Health Extension Program (HEP) is one of the most innovative community based health program launched by the Ethiopian Federal Ministry of Health to make health services accessible to rural communities by setting-out women Health Extension Workers (HEWs) in rural Health Posts. The HEWs are premised to provide basic, largely preventive, primary health services to rural villages and the program gives special attention to children and mothers. The objective of the study was to assess the contribution made by the Health Extension Workers in maternal and child health care service delivery in Dale district, southern Ethiopia.

**Methods:**

Using a community based cross-sectional data; the study assessed the status of mother’s health service utilization and estimated the role of HEWs in maternal and child health care delivery. Mothers of reproductive age (15–49), having at least one under-five age child, were eligible for the study. The total sample size was 617 and systemic random sampling method was used to select the study subjects from each randomly selected *kebeles* (lower administrative units). Structured questionnaire was applied to collect data through interviewing of the selected mothers and the data were analysed using SPSS version 16 statistical software.

**Results:**

Health Posts are important health care delivery settings and their share from the overall service delivery of ANC, Family planning and child treatment services were pivotal. However, overall service coverage of ANC (four and more visits), delivery and PNC services were low in the district as compared to the national status; and the input from the HEWs, in this regard, was unsatisfactory. The number of home visits was also inadequate for the necessary support of the mothers. The results of the multiple logistic regression indicated that mothers who listen to the radio (AOR 4.62; CI 1.66–12.85) and who had received information about the MCH services by HEWs (AOR 2.09; CI 1.06–4.14) were significantly associated with good MCH service utilization status.

**Conclusion:**

Health Extension Workers can improve their role in the MCH service delivery in the district by delivering appropriate information about the different available services to the mothers at the community and household level. All concerned bodies, including federal, regional and local governments should support the efforts of HEWs and need to address the challenges for poor performance areas of the HEWs in MCH service delivery.

## Background

In Ethiopia, pregnancy remains to be the leading cause of death of women in their reproductive years and the rate of under-5 and maternal mortality are among the highest in the world estimated at 67 deaths per 1000 live births and 412 deaths per 100,000 live births, respectively. Each year an estimated 22,000 women and 88,000 new-borns die from complications related to childbirth, mostly because of the large majority of the deliveries occur at home. In the last decade, the top four causes of maternal mortality were obstructed labor/uterine rupture, haemorrhage, hypertensive disorders of pregnancy and sepsis/infection [1, 2].

Moreover, Ethiopia is among the six countries that account for 50% of under-five child mortality globally, with 194,000 deaths every year. More than one third of the deaths are largely due to communicable diseases that could easily be prevented and treated using affordable and low-technology interventions. Nearly 68% of under-five deaths are attributed to pneumonia (21%); diarrhoea (14%); neonatal conditions such as prematurity (15%) and birth asphyxia (10%); measles (4%); malaria (2%) and HIV (2%) [3–5].

To address these maternal and child health problems and to increase access to health facility, the government of Ethiopia launched the Health Extension Program (HEP) in 2003; an innovative way of scaling up the delivery of basic and essential promotive, preventive and selected high impact curative health services targeting the household and community level by setting out women Health Extension Workers (HEWs) in rural Health Posts (HPs) [6–8].

The primary purpose of the HEP is to improve access and utilization of health care particularly for children and mothers. The program approach is based on the widely influential model known as the diffusion model, which holds that community behaviour can be changed step by step; training early adopters first, and then moving to the next group that is ready to change. Similarly, the HEP is designed to improve the health status of families, with their full participation, using local technologies and the community’s skill and knowledge [6, 7, 9].

A concerted effort by the government to expand the primary health care system and emphasize on preventive, promotive and basic curative health services resulted in a positive improvements in the health coverage and utilization; and consequently, maternal mortality ratio has shown a decreasing trend from the year 2000 estimate of 871 deaths per 100,000 live births to 412 deaths per 100,000 live births in 2016; and between 2000 and 2016, infant mortality has declined from 97 deaths per 1000 live births to 48 deaths per 1000 live births and under-five mortality from 166 deaths per 1000 live births to 67 deaths per 1000 live births [1, 10].

Based on the United Nations Inter-agency Group for Child Mortality Estimation (UN IGME) 2013 report, despite Sub-Saharan Africa’s relatively high rates of under-five mortality, there are signs of progress in the region. The rate of decline in under-five mortality has accelerated over time – from 0.8% a year over 1990–1995 to 4.1% a year over 2005–2012. According to the report, higher annual rates of reduction (5% per year) was observed in Ethiopia, which had reduced under-five mortality by more than a half from 1990 to 2012 (67%); and in all these progresses in the reduction of maternal and under-five child mortality rates, an important research question remains is that ‘How much the role of HEWs is noteworthy in the process of maternal and child health care service delivery?’ [11]. Previous published assessments of the roles of HEWs or the HEP were not inclusive of all major MCH components and the efforts made by the HEWs were not examined with the context of overall MCH service delivery [12–14]. In addition, the level of involvement of the HEWs in MCH service delivery was not assessed in the study area using a community based data. The objective of the study was to assess the level of contribution made by the Health Extension Workers in maternal and child health care service delivery in Dale district, southern Ethiopia.

## Methods

### Study design and setting

A community based cross-sectional survey was undertaken from May 24 to June 24, 2015 to assess the role of HEWs in MCH service delivery in rural villages of Dale district, Sidama zone, SNNPRS (Southern Nations, Nationalities and Peoples Regional State), Ethiopia. Dale district is one of the 19 districts in Sidama zone, and located 42 kms far from Hawassa town (capital city of SNNPRS) and 302 kms far from Addis Ababa town. It is divided into 3 urban and 33 rural *kebeles* (lower administrative units) and the total population of women within the reproductive age group in the area is estimated to be 61,639. Health Posts, which are headed and staffed by HEWs, represent the primary point of care.

### Study participants

Randomly selected mothers with under-five children who were willing and healthy enough to be interviewed were identified to participate in the survey. We employed the Statcalc sample size calculation for cross-sectional study using EPI-info version 3.5.4 to determine the sample size for our study. The total sample size was 617 and it was determined with an assumption of: 95% confidence interval, margin of sampling error tolerated - 5%, taking contraceptive prevalence rate of 25.8% (*P* = 0.25) for SNNPRS as key variable [15] and 5% non-response rate. Design effect of two was also considered because of the two sampling stages.

A two stage sampling technique was used to get representative mothers. Out of the total kebeles, 10 kebeles were selected randomly using simple random sampling (lottery) method and the required sample size for each selected kebele was taken according to the population size of each kebele. To select households with representative mothers, a map of each kebele was taken and the direction to start the first household was located using lottery method; then it was started from the first household and every fourth household was visited, by using a systematic random sampling method. If a home didn’t have the appropriate mother for the interview, the next home was visited until the sample required was obtained.

### Data collection procedure

Data was collected on mother’s utilization of family planning, ANC, delivery, PNC and child health care services. The questionnaire was initially developed in English and then translated to the local language, ‘Sidamigna’. Before the actual data collection, the questionnaire was pre-tested in other similar nearby district, to assure clarity of the concepts for respondents. The data were collected by twelve data collectors who had first degree and experience of doing questionnaire interviews; and training was given for the data collectors on details of the questionnaire. Responses of randomly selected questionnaires were re-checked by supervisors in the field to ensure rigor in the study.

### Data processing and analysis

The data were checked for its completeness; and were coded, entered, cleaned, explored and finally analysed using SPSS-version 16 statistical software. Descriptive statistics were used to summarize the data, and a bivariate analysis was carried out using binary logistic regression analysis to describe associations between exposure variables and MCH service utilization status of mothers with a significance level of *p*-value ≤0.05. In the multivariate analysis, potential independent variables were entered into the model; and a backward elimination procedure was applied and a *p*-value ≤0.05 was considered to identify predictors of good MCH service utilization.

### Operational definitions

Utilization of maternal and child health services were collected using the following variables:

- ***Family planning:*** If the mother has been using any contraceptive method during the interview period (current utilization).
- ***Antenatal care (ANC):*** If the mother attended a health facility for ANC, at least four times in her last pregnancy.
- ***Institutional delivery:*** If the mother gave birth in a health facility for her last child.
- ***Postnatal care (PNC):*** If the mother visited a Health Centre/Health Post within 24 h - 42 days of the birth of her last child.
- ***Immunization:*** If the mother had vaccinated her last under-five child for Penta 3 by validating mother’s verbal report with the records in the immunization card.
- ***Acute respiratory infection (ARI) treatment:*** If the youngest under-five child treated for symptoms of ARI in the 2 weeks preceding the survey.
- ***Diarrhoea treatment:*** If the youngest under-five child treated for diarrhoea in the 2 weeks preceding the survey.
- ***Fever treatment:*** If the youngest under-five child treated for fever in the 2 weeks preceding the survey.
- ***MCH service utilization:*** The dependent variable was computed by combining the utilization status of the above eight MCH services. Using the mean (2.63) as a cut-off point, we had categorized utilization of maternal health services into two categories. Mothers who had utilized three and more maternal health services were defined as having ‘Good utilization’ of MCH services, while those who had utilized less than three were considered as having ‘Poor utilization’ of MCH services.
- ***Information delivery by HEWs about MCH services:*** Mothers were asked whether they have information or not about the eight MCH services given by HEWs. Mothers who had information on at least two MCH services were defined as having ‘good information’, while those who had no information or had information for only one MCH service were considered as having ‘poor information’.
- ***Counselling and follow up:*** If the mother took counselling and followed for continuous utilization of at least one MCH service.
- ***Service delivery approach:*** There were six items to measure the different perceived perspectives of the MCH service delivery approach. These were, giving complete explanation about the service/health problem, friendly approach during service delivery, making helpful suggestions, discuss treatment/service options, giving of the health service/treatment with respect; and helping the mother to understand her own/her child’s health condition. Two and more positive answers were considered as the mother was satisfied with the service delivery approach.
- ***A model family:*** A family which has fulfilled and implemented all packages of the HEP.

## Results

A total of 613 mothers with under-five children, were interviewed and the response rate was 99.4%. The majority of the respondents was found to be Sidama by ethnicity, 583 (95.1%), Protestant by religion (83.4%), and 32% were in the age group of 25–29 years during the delivery of their last child. Furthermore, the majority of the respondents were married accounting 94.4%; and the mean age of the interviewed mothers was 28 years. Of the total mothers, 83.4% were house wives/not having paid work and 12.2% were farmers. Regarding the educational background of the mothers, 23.4% of the mothers didn’t ever attended school and 63.6% of them attended only primary education. Most of the respondents (83%) had no access to listen to the radio and 54% of the mothers were graduated as a model household (Table 1).

**Table 1:** Socio-demographic characteristics of mothers in Dale district, southern Ethiopia, 2015

Variables	Category	Frequency	Percent (%)
Maternal age	15–19	85	14.5
	20–24	183	31.3
	25–29	187	32.0
	30–34	86	14.7
	35–39	30	5.1
	> 40	14	2.4
Mother’s educational status	Illiterate	143	23.4
	Primary education	388	63.6
	Secondary education	68	11.1
	Higher education	11	1.8
Marital status	Married	576	94.4
	Widowed	21	3.4
	Divorced	13	2.1
Age of the index child (months)	Neonate	58	9.4
	1–6	176	28.7
	7–11	88	14.3
	12–23	99	16.1
	24–59	192	31.3
Ethnicity	Sidama	583	95.1
	Amhara	14	2.3
	Other	16	2.7
Religion	Protestant	511	83.4
	Orthodox	22	3.6
	Catholic	63	10.3
	Muslim	17	2.8
Occupational status	Housewife	511	83.4
	Farmer	75	12.2
	Other	27	4.4
Listen to the radio	Yes	101	17
	No	492	83
Graduated as a model family	Yes	331	54
	No	282	46

### MCH service utilization status

Most of the mothers (95.4%) were not pregnant during the survey and want to have a baby (60.9%). Of the total mothers, 58.9% had a history of ANC follow-up during their last pregnancy and 14.3% of them had attended at least the recommended four visits. A large proportion of mothers (89.6%) who attended ANC follow-up had received tetanus toxoid injections and about half of them had received only one injection. Out of the total mothers, 22.5% of them had delivered their last child at health institutions and more than three fourth of them gave birth at home. Among the mothers who gave birth at health institutions, 26.7% of them had delivered their last child by caesarean section.

According to the mothers’ report, more than one third of the mothers (37%) had attended PNC service within 6 weeks after delivery. 70.5% of the mothers were currently using family planning methods and 40.8% of the mothers had vaccinated their child for Penta 3 vaccine. Regarding seeking care for under-five children with a history of illness, 52.3%, 38.7% and 39.3% were treated for diarrhoea, acute respiratory infections and Acute Febrile Illnesses (AFI), respectively (Table 2).

**Table 2:** MCH service utilization status of mothers in Dale district, southern Ethiopia, 2015

MCH services	Service utilization status	Frequency	Percent (%)
Antenatal care	Yes	88	14.3
	No	525	85.6
Institutional delivery	Yes	138	22.5
	No	475	77.5
Postnatal care	Yes	227	37
	No	386	63
Family planning	Yes	428	70.5
	No	179	29.5
EPI	Yes	250	40.8
	No	362	59.2
Diarrhoea treatment for <5 children	Yes	69	52.3
	No	63	47.7
Acute Respiratory Infection (ARI) treatment	Yes	63	38.7
	No	100	61.3
Acute Febrile Illness (AFI) treatment	Yes	77	39.3
	No	119	60.7

### Role of Health Extension Workers in MCH service delivery

Of the total mothers, 428 (69.82%) mothers visited Health Post for at least one health service of their own or their last child. Health Post accounts’ for 50% of ANC follow up, 55.6% of family planning and 3.2% of delivery services; and 27.5% and 48% of diarrhoea and AFI treatment services, respectively. Private clinics also have an important share in the delivery of MCH services, accounting 12.5% of ANC follow up, 25.9% of AFI treatment and 5.1% of family planning services (Table 3).

**Table 3:** Place of receiving MCH services in Dale district, southern Ethiopia, 2015

MCH services	Place where the service utilized	Frequency	Percent (%)
Antenatal care	Health Post	44	50
	Health centre	29	32.9
	Hospital	4	4.5
	Private clinic	11	12.5
Delivery	Home	475	77.5
	Health Post	20	3.2
	Health centre	75	12.2
	Hospital	29	4.7
	Private clinic	14	2.2
Family planning	Health Post	238	55.6
	Health centre	165	38.5
	Hospital	3	0.7
	Private clinic	22	5.1
Diarrhoea treatment for <5 children	Health Post	19	27.5
	Health Centre	43	62.3
	Hospital	7	10.1
Acute Febrile Illness (AFI) treatment	Health Post	37	48
	Health Centre	15	19.4
	Hospital	5	6.5
	Private clinic	20	25.9

**Fig. 1:**
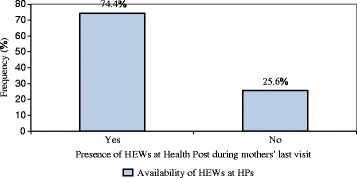
Availability of Health Extension Workers during mothers’ last Health Post visit in Dale district, southern Ethiopia, 2015. (Blue square) Availability of HEWs at HPs

**Fig. 2:**
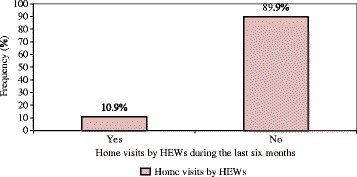
Home visits by Health Extension Workers (HEWs) during the last 6 months in Dale district, southern Ethiopia, 2015. (Red square) Home visits by HEWs

Out of the mothers who knew MCH services are given in Health Posts, the following proportion of mothers knew that the services are available in HPs; 56.8% - family planning, 51.5% - ANC, 44.4% - delivery, 45.6% - PNC, 80.5% - immunization, 60.2% - growth monitoring and 45.5% - sick baby care services (Table 4). Moreover, 195 (74.4%) of the mothers also described that HEWs were available at HPs during their last visit for MCH services (Fig. 1). Within the preceding 6 months of the survey, 10.9% of the mothers were visited by HEWs and received MCH services at their home (Fig. 2).

**Table 4:** Information about the different MCH services given by HEWs in Dale district, southern Ethiopia, 2015

Information about the MCH services	Category	Frequency	Percent (%)
Knew family planning as MCH service	Yes	184	56.8
	No	91	28.1
Knew ANC as MCH service	Yes	132	51.5
	No	124	48.5
Knew institutional delivery as MCH service	Yes	110	44.4
	No	138	55.6
Knew PNC as MCH service	Yes	117	45.6
	No	139	54.3
Knew immunization as MCH service	Yes	210	80.5
	No	51	19.5
Knew growth monitoring as MCH service	Yes	159	60.2
	No	105	39.7
Knew sick baby care as MCH service	Yes	117	45.5
	No	140	54.5
Knew diarrhoea treatment as MCH service	Yes	106	39
	No	166	61

Based on the mothers’ report; 61.1% of the mothers indicated that they had gotten a complete explanation of her own/child’s health condition from the HEWs, 56.7% and 54.8% stated that the HEWs were friendly and helped to understand their own/their child’s health condition, respectively. Moreover, 50.5% of the mothers reported that HEWs had made helpful suggestions about the prevention of maternal/child illnesses (Table 5).

**Table 5:** Service delivery approach by HEWs in Dale district, southern Ethiopia, 2015

Service delivery approach	Category	Frequency	Percent (%)
Gave complete explanation	Yes	113	61.1
	No	72	38.9
Friendly	Yes	106	56.7
	No	81	43.3
Made helpful suggestions	Yes	92	50.5
	No	90	49.5
Treated with respect	Yes	94	51.1
	No	90	48.9
Discuss treatment options	Yes	99	52.9
	No	87	46.5
Help me to understand my/child’s health condition	Yes	102	54.8
	No	84	45.2

### Determinant factors of good MCH service utilization status

Determinant factors of good MCH service utilization were assessed with the perspective of HEW’s key roles and activities. Selected socio-demographic variables and variables which indicate major activities of the HEWs were assessed to determine their association with good service utilization status. Mother’s educational status, listening to the radio, information delivery by HEWs about MCH services and home visits in the last 6 months by HEWs were significant in the bivariate analysis with *p*-value ≤0.05 (Table 6).

**Table 6:** Results from bivariate analysis of different factors for good MCH service utilization status in Dale district, southern Ethiopia, 2015

Variables	Category	Frequency	COR (95% CI)	*P*-value
Maternal age	15–19	85	1	
	20–24	183	3.30 (1.01, 10.74)	0.06
	25–29	187	1.78 (0.57, 5.51)	0.31
	30–34	86	1.98 (0.64, 6.13)	0.23
	35–39	30	1.97 (0.61, 6.38)	0.25
	> 40	14	1.20 (0.32, 4.47)	0.78
Mother’s educational status	Illiterate	143	1	
	Primary education	388	0.90 (0.53, 1.51)	0.69
	Secondary education	68	7.55 (0.95, 59.61)	0.05
	Higher education	11	0.44 (0.30, 0.66)	< 0.001^a^
Marital status	Married	576	1	
	Widowed	21	1.26 (0.53, 3.72)	0.49
	Divorced	13	1.17 (0.38, 2.87)	0.61
Ethnicity	Sidama	583	1	
	Amhara	14	1.21 (0.26, 3.94)	0.53
	Other	16	1.69 (0.54, 4.42)	0.38
Religion	Protestant	511	1	
	Orthodox	22	0.41 (0.04, 4.22)	0.45
	Catholic	63	0.19 (0.01, 2.15)	0.18
	Muslim	17	0.29 (0.02, 3.45)	0.33
Listen to the radio	Yes	101	2.35 (1.48, 3.71)	< 0.001^a^
	No	492	1	
Information delivery by HEWs	Yes	269	2.39 (1.72, 3.32)	< 0.001^a^
	No	345	1	
Home visits by HEWs	Yes	67	2.12 (1.23, 3.66)	< 0.01^a^
	No	544	1	
Availability of HEWs at HPs	Yes	195	1	
	No	67	0.64 (0.36, 1.12)	0.11
Service delivery approach	Positive	101	1.35 (0.75, 2.42)	0.31
	Negative	88	1	
Graduated as a model family	Yes	331	1	
	No	282	0.76 (0.45, 2.39)	0.26

^a^Statistically significant at p < 0.05

In order to identify predictors of good MCH service utilization, the possible confounding effect was controlled using multiple logistic regression analysis; and found that mothers who listen to the radio (AOR 4.62; CI 1.66–12.85) and who had received information about MCH services by HEWs (AOR 2.09; CI 1.06–4.14) were more likely to use MCH services than mothers who had not (Table 7).

**Table 7:** Determinant factors of good MCH service utilization status in Dale district, southern Ethiopia, 2015

Variables	Category	Frequency	AOR (95% CI)	*P*-value
Mother’s educational status	Illiterate	143	1	
	Primary education	388	2.37 (0.72, 7.78)	0.15
	Secondary education	68	4.77 (0.00,)	0.99
	Higher education	11	0.52 (0.23, 1.18)	0.12
Information delivery by HEWs	Yes	269	2.09 (1.06, 4.14)	0.03^a^
	No	345	1	
Listen to the Radio	Yes	101	4.62 (1.66, 12.85)	0.003^a^
	No	492	1	

^a^Statistically significant at *p* < 0.05

## Discussion

The coverage of MCH service utilization in Dale district had decreased when compared to the national status. For instance, when we take a look at ANC follow up and delivery service coverage; ANC follow up (at least four visits) – it was 14.3% in the study area and 32% in EDHS 2016; and institutional delivery – 22.5% and 28% in our survey and EDHS 2016, respectively. This may be due to less effect of the HEWs in the district or the reason that the national cumulative report sometimes may not reflect actual district level performances [1].

Mothers had received services primarily from Health Posts for family planning, ANC follow up and AFI treatment services and Health Centres were dominant places of receiving care for institutional delivery, PNC and diarrhoea treatment services. This finding is comparable to other studies done in northern Ethiopia [12, 13].

According to our study, listening to the radio and information delivery about MCH services by HEWs had significant association with good MCH service utilization status. The study conducted in Tigray region also showed that listening to the radio had significant association with good MCH service utilization status [12]. In addition, an interventional study conducted in Kenya from 2008 to 2009 revealed that Community Health Workers’ (CHWs) information delivery to mothers about MCH services, through monthly dialogue with the community, had improved proportion of mothers who attend ANC follow up, family planning and immunization services. Furthermore, the study had reported a progress in use of both PNC and institutional delivery services [16].

In support with our finding, ACCESS/MCHIP evaluation survey conducted in Nigeria regarding the program implementation which uses CHWs as a channel for awareness creation on family planning, ANC follow up, institutional delivery and immunization services; showed there was improvement in MCH service utilization [17]. Moreover, a community-based intervention in Tanzania was designed and implemented by 50 trained safe motherhood promoters (SMPs) with a focus to promote early and complete antenatal care visits; and skilled delivery. The result showed that early ANC booking and deliveries with a skilled attendant had increased significantly [18].

Though our study highlights several potential needs to improve the role of HEWs in the delivery of MCH services, it has certain limitations. First, as the study is cross-sectional design, no causal conclusions can be drawn. Second, the study relies on self-reported data and a potential recall bias could have been present. Third, the study lacks reference in determining ‘Good’ and ‘Poor’ MCH service utilization cut-off points.

## Conclusions

The primary purpose of the study was to assess the role of HEWs in promoting and expanding MCH services, which in turn helps to improve child and maternal health outcomes. The result showed that Health Posts are important health care delivery settings and their share from the overall service delivery of ANC, Family planning and child treatment services were pivotal and HEWs can improve their role in the MCH service delivery in the district by delivering appropriate information about the different available services to the mothers at the community and household level.

Despite these facts, overall service coverage of ANC (four and more visits), delivery and PNC services were low in the district as compared to the national status (EDHS 2016); and the input of the HEWs, in this regard, was unsatisfactory. The number of home visits was also inadequate for the necessary support of the mothers. Therefore, in order to address these gaps and improve the role of HEWs in the delivery of MCH services, all concerned bodies, including federal, regional and local governments should support the efforts of HEWs and need to address the challenges for poor performance areas of the HEWs in MCH service delivery.

The effect of the program on maternal and child health as well as the demand and supply side factors that influence MCH service utilization, should be studied in greater depth in future studies in order to improve the performance of the HEP continuously.

## Abbreviations

AFI: Acute Febrile Illness
ANC: Ante-natal care
AOR: Adjusted odds ratio
CHWs: Community Health Workers
CI: Confidence interval
COR: Crude odds ratio
EDHS: Ethiopian Demographic and Health Survey
HEP: Health Extension Program
HEWs: Health Extension Workers
HPs: Health Posts
MCH: Maternal and child health
PNC: Post-natal care
